# Effects of Degrees of Degeneration on the Electrical Excitation of Human Spiral Ganglion Neurons Based on a High-Resolution Computer Model

**DOI:** 10.3389/fnins.2022.914876

**Published:** 2022-07-06

**Authors:** Albert M. Croner, Amirreza Heshmat, Anneliese Schrott-Fischer, Rudolf Glueckert, Werner Hemmert, Siwei Bai

**Affiliations:** ^1^Department of Electrical and Computer Engineering, Technical University of Munich, Munich, Germany; ^2^Munich Institute of Biomedical Engineering, Technical University of Munich, Garching, Germany; ^3^Laboratory for Inner Ear Biology, Medical University of Innsbruck, Innsbruck, Austria

**Keywords:** cochlear implant, computational model, human, spiral ganglion neurons, neural degeneration, ectopic activation, personalized model, deep insertion

## Abstract

After hearing loss retrograde degeneration of spiral ganglion neurons (SGNs) has been described. Studies modeling the effects of degeneration mostly omitted peripheral processes (dendrites). Recent experimental observations indicated that degenerating SGNs manifested also a reduced diameter of their dendrites. We simulated populations of 400 SGNs inside a high resolution cochlear model with a cochlear implant, based on μCT scans of a human temporal bone. Cochlear implant stimuli were delivered as biphasic pulses in a monopolar configuration. Three SGN situations were simulated, based on our previous measurements of human SGN dendrites: (A) SGNs with intact dendrites (before degeneration), (B) degenerating SGNs, dendrites with a smaller diameter but original length, (C) degenerating SGNs, dendrites omitted. SGN fibers were mapped to characteristic frequency, and place pitch was estimated from excitation profiles. Results from degenerating SGNs (B, C) were similar. Most action potentials were initiated in the somatic area for all cases (A, B, C), except for areas near stimulating electrodes in the apex with intact SGNs (A), where action potentials were initiated in the distal dendrite. In most cases, degenerating SGNs had lower thresholds than intact SGNs (A) (down to –2 dB). Excitation profiles showed increased ectopic activation, i.e., activation of unintended neuronal regions, as well as similar neuronal regions excited by different apical electrodes, for degenerating SGNs (B, C). The estimated pitch showed cases of pitch reversals in apical electrodes for intact SGNs (A), as well as mostly identical pitches evoked by the four most apical electrodes for degenerating SGNs (B, C). In conclusion, neuronal excitation profiles to electrical stimulation exhibited similar traits in both ways of modeling SGN degeneration. Models showed degeneration of dendrites caused increased ectopic activation, as well as similar excitation profiles and pitch evoked by different apical electrodes. Therefore, insertion of electrodes beyond approximately 450° may not provide any benefit if SGN dendrites are degenerated.

## 1. Introduction

In normal hearing humans, a sound consisting of a single frequency will excite neurons in a limited and specific area within the cochlea, with high frequencies exciting neurons more in the base, and low frequencies exciting neurons more in the apex (von Békésy, 1960). This tonotopic arrangement of the cochlea is also exploited for cochlear implants (CIs), where multiple electrodes are placed in the cochlea at different positions. The coding strategy of the CI converts sound which is recorded by a microphone behind the ear into stimulation currents delivered to individual electrodes, which in turn stimulate distinct neuron populations (Loizou, 1999; Wilson, 2008). Overall, CIs are able to restore speech understanding in otherwise profoundly hearing impaired patients to a surprisingly high degree, which makes them the most successful neuroimplants today. However, the extent to which hearing fidelity is restored in CI patients still differs, with hearing performance varying strongly between individual subjects (Blamey et al., 1996; Holden et al., 2013). There are multiple possible reasons contributing to this variance in performance. One such reason may be that every human has an individual cochlea, i.e., when two inner ears are implanted with the same CI, differing cochlear geometries may lead to differences in electric current flow. This is especially relevant as electric stimulation impedes the precision of neural excitation compared to acoustic, i.e., with electric stimulation it is not possible to perfectly exploit the tonotopic arrangement of the cochlea. Imperfect exploitation of the tonotopic map manifests as e.g., a low number of independent frequency channels in CI stimulation (Croghan et al., 2017), or stimulation of unintended neuronal regions (e.g., “cross-turn” stimulation, Kalkman et al., 2014; “tip-shifts,” Nelson et al., 2008). Another reason may lie within the spiral ganglion neurons (SGNs) responsible for the transmission of sound information, which may degenerate or die (Glueckert et al., 2005) and thereby affect the auditory information transmitted to the brain.

In order to estimate how cochlear geometry and state of SGNs, i.e., their morphology, influence the performance of CI subjects in experiments, it would be necessary to measure both cochlear geometry and SGN morphology. The former can be measured to a limited degree in living patients, e.g., using CT (Nogueira et al., 2016). However, measurements in living patients, especially of implanted cochleae, will only yield rough measures such as the height and diameter of the cochlea. Higher resolution can only be measured post-mortem, e.g., using slice preparations or μCT scans. Therefore, as cochlear measurements in living patients are severely limited, several volume conduction models of human cochleae were developed over the years. Cochlea models employed to simulate CI stimulation started out as manually created geometries, e.g., a model of an unrolled tube (Finley et al., 1990), or of a coiled, tapering tube (Frijns et al., 2001; Hanekom, 2001), including basic structures such as cochlear scalae. Those manually created geometries were later employed to generate “personalized” models, i.e., by fitting the model geometries to measurements of actual human cochleae, such as height, diameter, or post-mortem mid-modiolar cross-sections (Kalkman et al., 2014; Malherbe et al., 2016; Nogueira et al., 2016). Those models, while including some degree of personalization, were still based on simple geometries. A more recent development is the creation of models based on high-definition μCT scans of post-mortem human cochleae, including segmentation of individual structures. As creating such detailed models is especially labor intensive, there exist only a small number of those to date. Nevertheless, they offer the advantage of including fine details of structures present in physical cochleae, which influence the current spread in the cochlea under CI stimulation (Bai et al., 2019; Potrusil et al., 2020).

Cochlear volume conduction models allow for examination of current spread within the cochlea, but do not give information about whether the actual neurons in the auditory nerve are excited. Therefore, they are generally combined with multi-compartment neuron models, representing type I SGNs. However, SGNs may manifest different morphologies which need to be taken into account, especially in profoundly hearing impaired subjects who are candidates for a CI implantation. One hearing-loss-induced change in SGN morphology is the retrograde degeneration of peripheral neuronal processes, caused by deafferentiation to inner hair cells. Such neural degeneration has been observed in both animals (Bichler et al., 1983; Spoendlin, 1984; Wise et al., 2017) and humans (Nadol, 1990; Glueckert et al., 2005; Linthicum and Fayad, 2009; Rask-Andersen et al., 2010). Neural degeneration has, therefore, been incorporated in several models, in most cases by modeling neuron populations with both an intact peripheral process and a completely missing peripheral process (Rattay et al., 2001, 2013; Briaire and Frijns, 2006; Smit et al., 2008, 2010; Snel-Bongers et al., 2013; Kalkman et al., 2014; Malherbe et al., 2015; Potrusil et al., 2020). Intact and missing peripheral processes are the extremes of neural degeneration of the auditory nerve, excluding the actual death of SGNs. It is, however, not clear how the degenerative process proceeds in between those two extremes, especially for humans. A possible intermediate degenerative state is the shrinking of peripheral processes' diameter, as described by Heshmat et al. (2020).

In this study, we present a detailed model of the human inner ear, consisting of a high resolution finite element (FE) volume conduction model of a human cochlea and a population of modeled type I SGNs. The FE cochlea model was based on μCT scans of an implanted human cochlea and included reconstructed nerve fiber paths (Bai et al., 2019). Neuron populations were based on the multi-compartment model from Rattay et al. (2013) and modeled 400 auditory nerve fibers (ANFs) spread out along the tonotopic axis. With the modeled neuron population, differences in excitation behavior based on different degenerative stages were investigated, including an intermediate stage with thin peripheral processes. In this study, the peripheral process of a modeled ANF is denoted as “dendrite” and the central process as “axon.”

## 2. Methods

### 2.1. Reconstruction of the Cochlea Model

**Figure 1:**
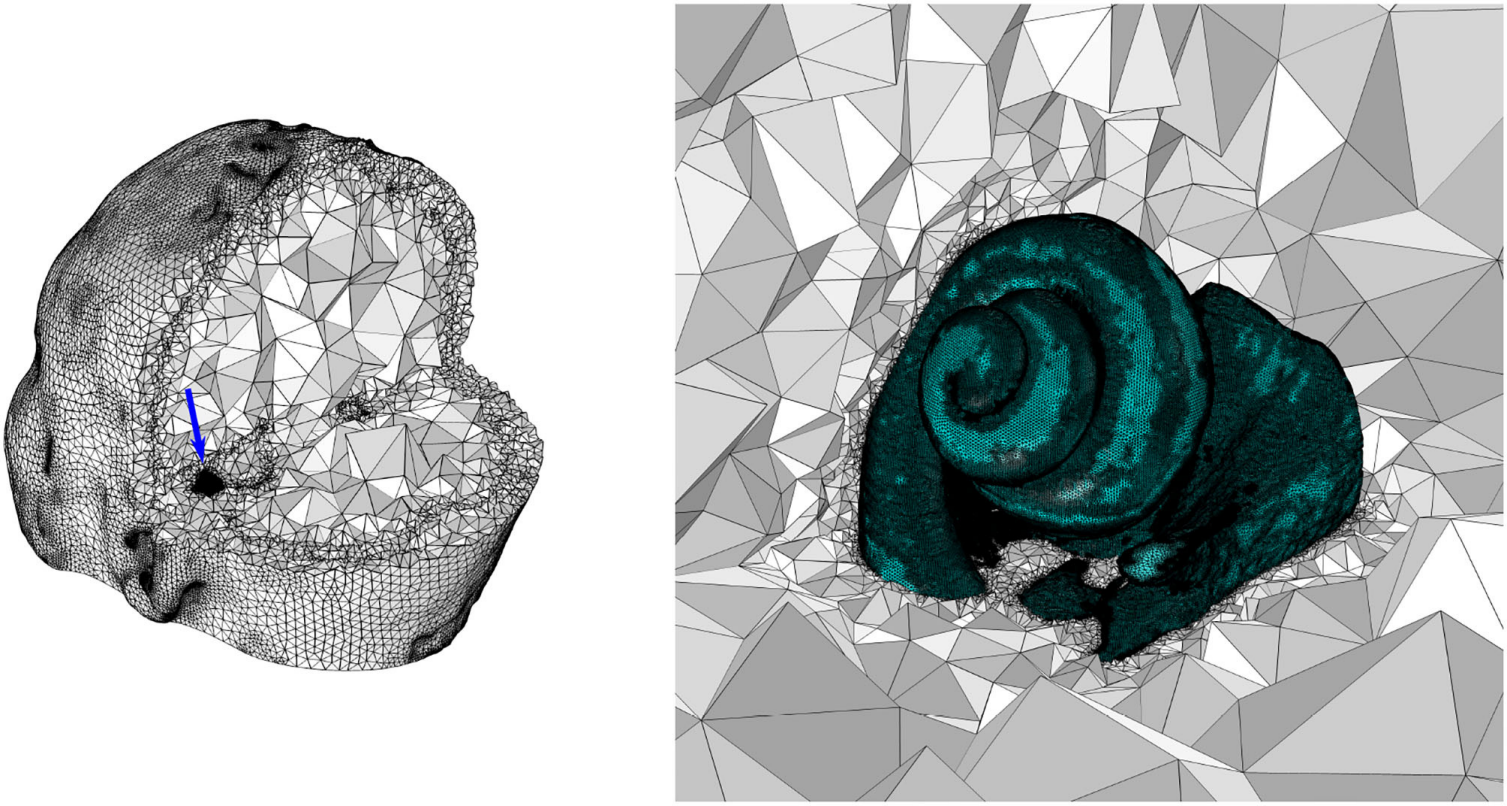
Left: A dissected view of the FE mesh of the whole head model. The blue arrow indicates the location of the cochlea. Right: A zoomed-in view of the cochlea (and the nerve), indicated by the blue arrow in the left image.

**Figure 2:**
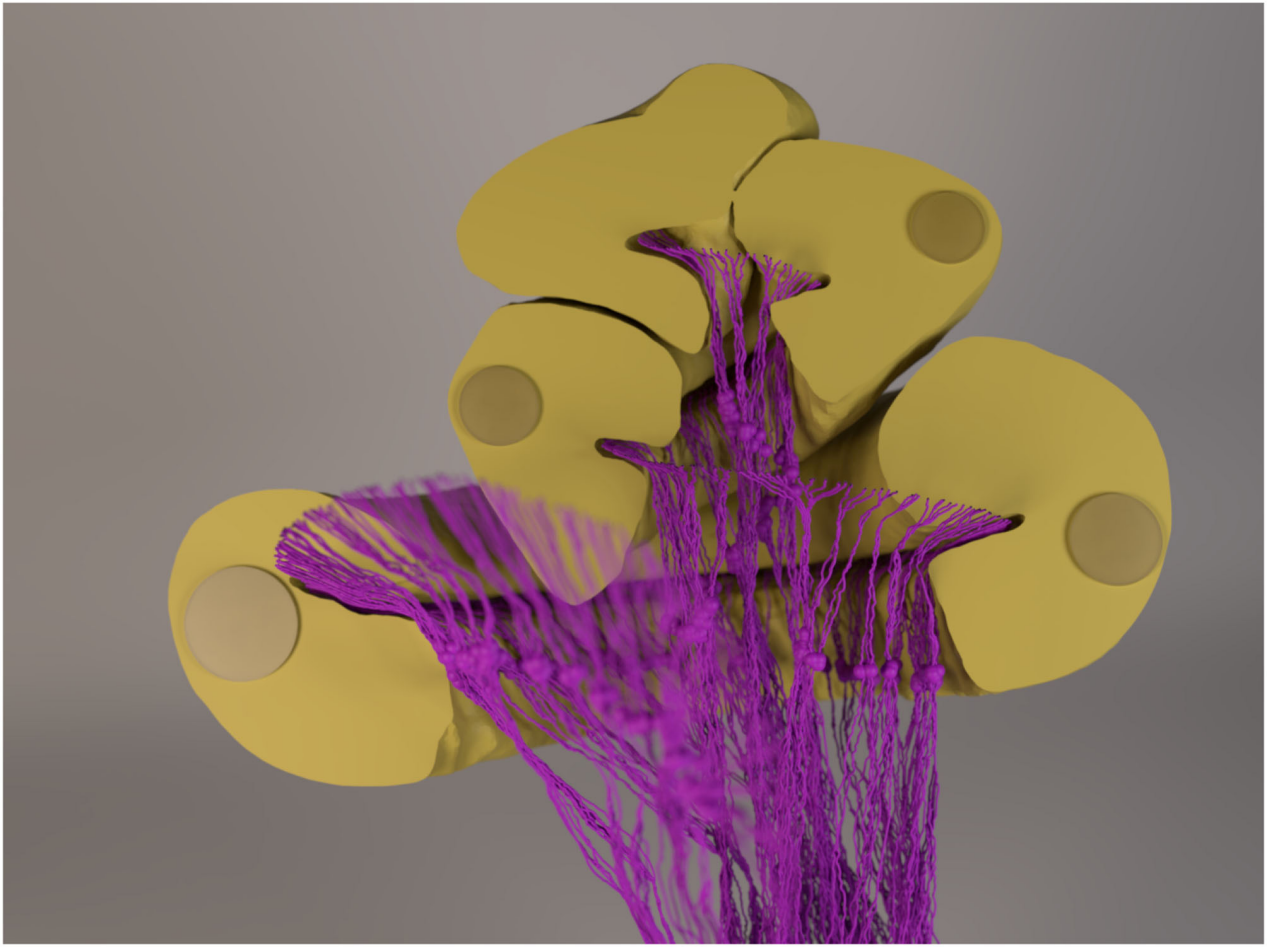
A rendered image of the 3D cochlea model and the neurons with 1.5-mm-long dendrites. The cochlear canal, as well as the implanted electrode, was cut to provide a better view of the neurons. The “out-of-focus” neurons in the image sit in front of the cut-plane. Note that the diameter of the fibers (and the somata) is amplified for visualization purposes, i.e., they are not to scale.

The FE cochlea model with an implanted electrode array was reconstructed from a set of μCT scans of a human cadaveric temporal bone. The implanted cochlea model was then placed in a human head model at the petrous part of the left temporal bone, which was necessary to correctly place the CI ground electrode. This resulted in a FE model with 21,937,778 volumetric mesh elements, shown in Figure 1. The trajectories of 400 ANFs spanning from the base to the apex of the cochlea were reconstructed based on the FE mesh of the auditory nerve. The lengths of the reconstructed neuron paths ranged from 5.5 to 8.2 mm. Neuron paths are illustrated in Figure 2. The complete FE model was subsequently imported into COMSOL Multiphysics (COMSOL AB, Sweden) for calculating the electric potential *V* in a volume conduction model. The CI electrode array was modeled representing a MED-EL (Innsbruck, Austria) Standard electrode, which comprises 12 pairs of electrode contacts (≡ 12 electrodes), and was based on the geometry of a dummy electrode array physically inserted into the temporal bone sample. The MED-EL Standard electrode is considered a lateral wall electrode array. Note that the electrode array inserted into our preserved cadaveric temporal bone punctured the basilar membrane at approximately 270° into the cochlea, thereby traversing from the scala tympani into the scala vestibuli. As consequence, the modeled electrode array was situated in the scala vestibuli from electrode 6 upwards. Traversal of electrode arrays into the scala vestibuli is regularly observed in implanted cochleae, with arrays either partially or entirely located in scala vestibuli (e.g., approximately 25% of cochleae, Wardrop et al., 2005; approximately 5% of cochleae, O'Connell et al., 2016; Risi, 2018). The presence of scala traversal may, however, be a limitation of the model. It is yet to be studied whether the traversal would influence modeling outcomes.

**Figure 3:**
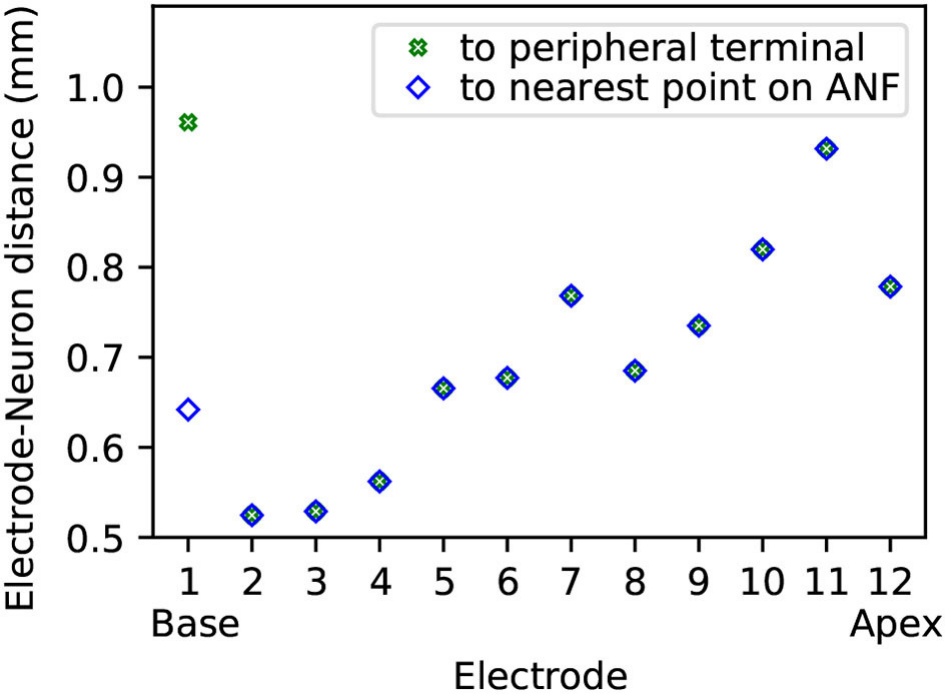
Electrode-neuron distance for each of the twelve electrodes in the model. The distance is displayed both as distance to the closest point along the beginning of the reconstructed neuron paths (≡ modeled peripheral terminals), and as distance to the closest point along the entire length of all reconstructed neuron paths (≡ modeled ANFs).

Electrode contact pairs were numbered with the most basal electrode pair as “electrode 1,” and the most apical electrode pair as “electrode 12.” The CI stimulation scheme in this study was monopolar: the current-controlled stimulus was delivered from a single electrode, with the ground electrode placed extracochlearly on the left temporal bone of the skull. Electrode-neuron distance, i.e., the shortest distance from an electrode to either the beginning of a neuron path or to the nearest point along a neuron path, ranged from approximately 0.5 mm to 1 mm, as depicted in Figure 3. For the purpose of calculating electrode-neuron distance, the coordinate of an electrode was determined as the center point between the two electrode contacts of a pair. Finally, the electric potentials on all points along the ANF paths were extracted from the FE simulation.

A detailed description of the reconstruction and electrical properties of the FE model, as well as the reconstruction of neuron trajectories, can be found in Bai et al. (2019). Electrode dimensions and the conductivity values of the FE model can also be found in Supplementary Table S1, available in the online version of this article.

### 2.2. Multi-Compartment Neuron Model

A multi-compartment model adapted from Rattay et al. (2013, 2001) was implemented. Our ANF model followed the description in the original publication except for the morphology of dendrites and axons. Simulations were run with a population of 400 ANFs. Population simulation was done by simulating the multi-compartment neuron model with extracellular electric potential values obtained from the FE model at the 400 previously reconstructed neuronal paths.

The model from Rattay et al. (2013) was originally designed with a 1 μm dendrite diameter and a 2 μm axon diameter. However, histograms from our recent measurements on dendrite diameter exhibited a unimodal distribution for normal hearing cases with a maximum at 2 μm diameter, and a bimodal distribution for patients with hearing loss, peaking at 0.5 and 2 μm diameters (Heshmat et al., 2020). Therefore, we used a dendrite diameter of 2 μm in our model to represent “intact” dendrites. Based on the ratio of $\frac{diameter_{axon}}{diameter_{dendrite}}=2$ in Rattay et al. (2013), the axon in our model was, thus, implemented with a diameter of 4 μm; this also lies around the upper limit of type I SGN axon diameters observed (Arnesen and Osen, 1978; Nadol, 1990; Nadol et al., 1990). In addition to “intact,” non-degenerated dendrites, we also modeled two further degenerative states. Based on the measurements with hearing-loss cases, a “partially degenerated" dendrite was modeled with a diameter of 0.5 μm, and a “completely degenerated" dendrite was modeled by omitting the entire dendrite, modeling the neurons with only soma and axon. In both degenerated cases, the axon diameter was kept identical to the “intact" case, with a diameter of 4 μm. Heshmat et al. (2020) also included measurements on myelin thickness in dendrites (peaking at 0.6 μm for normal hearing, and at 0.6 μm and 0.15 μm for hearing loss), which were implemented in our model as well, with “intact" dendrites having a myelin thickness of 0.6 μm, and “partially degenerated" dendrites a myelin thickness of 0.15 μm.

**Figure 4:**
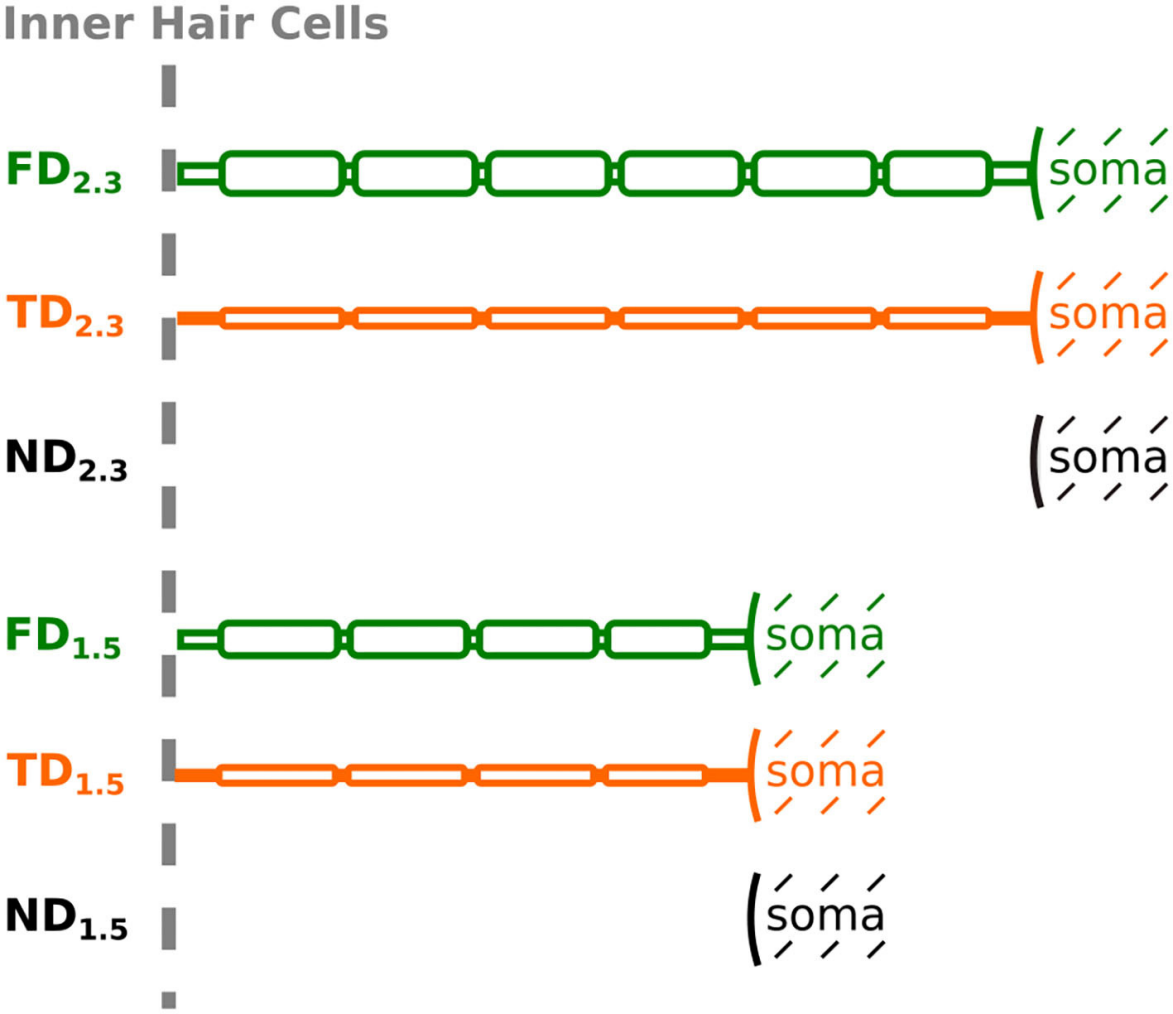
Schematic representation of an ANF for different degenerative states and dendrite lengths, depicted up to soma (not to scale). Degenerative state is indicated by name [FD, Full (intact) Dendrite; TD, Thin Dendrite; ND, No Dendrite] and dendrite length is indicated by the subscript.

Regarding dendrite length, we implemented two versions, as in Rattay et al. (2001): one with a dendrite length of 2.3 mm, and the other with a dendrite length of 1.5 mm, to account for shorter dendrites in the middle turn of the cochlea and longer dendrites in the base and apex (Potrusil et al., 2020). In a single simulation run, dendrite lengths were kept uniform for all 400 neurons, whereas the axon lengths were adapted by adding or removing node-internode pairs so that all fibers ended with an internode at the end of their reconstructed paths. Intact ANFs started with their peripheral terminal (first dendritic compartment) at the beginning of the reconstructed paths, i.e., in the spiral lamina attached at the level of inner hair cells. As described above, three degenerative states were implemented for the neuron population: “intact” dendrites, “partially degenerated” dendrites with narrower diameter, and “completely degenerated” dendrites with the entire dendrite removed. Hence, we have the following six combinations, also illustrated in Figure 4:

- FD_2.3_: **F**ull (intact) **D**endrite. 2.3 mm dendrite length and 2 μm dendrite diameter.
- TD_2.3_: **T**hin **D**endrite. 2.3 mm dendrite length and 0.5 μm dendrite diameter.
- ND_2.3_: **N**o **D**endrite. Soma, as well as the axon, positioned at the same location as FD_2.3_ and TD_2.3_, therefore, the first neuronal compartment (soma) offset by 2.3 mm from the beginning of the path.
- FD_1.5_: **F**ull (intact) **D**endrite. 1.5 mm dendrite length and 2 μm dendrite diameter.
- TD_1.5_: **T**hin **D**endrite. 1.5 mm dendrite length and 0.5 μm dendrite diameter.
- ND_1.5_: **N**o **D**endrite. Soma, as well as the axon, positioned at the same location as FD_1.5_ and TD_1.5_, therefore, the first neuronal compartment (soma) offset by 1.5 mm from the beginning of the path.

Parameters of the multi-compartment neuron model can be found in Supplementary Table S1, available in the online version of this article.

### 2.3. Processing

Neuron models were implemented in Python 3.5, using the Brian2 package (Goodman and Brette, 2009), and simulations were conducted on a computing cluster in parallel using the Thorns package (Rudnicki, 2022). Differential equations were solved using an exponential Euler method with a 1 μs timestep. The current stimulus used for neuronal stimulation was a single biphasic (cathodic-first) pulse with 40 μs/phase. For each of the six simulated degenerative states and 12 stimulation electrodes, the minimum current necessary to elicit an action potential (AP) in at least a single fiber, i.e., threshold, and all 400 fibers was determined. The range spanned between those two current values would then define the electric dynamic range (EDR). Afterward, each degenerative state and stimulation electrode was simulated with 80 current stimuli linearly spaced along within the EDR. For all simulations, voltage traces were saved and subsequently evaluated to determine the AP initiation site for all amplitudes.

Initial results were the “excitation profiles,” which show when ANFs were excited, i.e., generated an AP, for a neuron population within its entire EDR. To compare excitation profile shapes of different degenerative states, the difference of individual fiber thresholds in dB was computed, using Equation 1,


(1) $$\begin{array}{r} 20\cdot log_{10}\left(\frac{thr_{a,i}}{thr_{b,i}}\right) \end{array}$$

where *thr* is the threshold of an individual fiber, *a* and *b* denote the degenerative state, i.e., FD or TD for *a*, and ND for *b*, and *i* denotes the individual fiber, numbered from 1 to 400.

To represent characteristic frequencies (CF) of individual fibers, ANFs were mapped to frequencies using the Greenwood frequency map (Greenwood, 1990). For mapping purposes, relevant fiber coordinates were the positions of peripheral tips of the fibers, i.e., the starting point of the peripheral terminals. Fiber tip coordinates were transformed into 1-dimensional coordinates, i.e., describing only the distance along the fiber tip trajectory, with the most basal fiber (fiber 1) denoting the starting point. 1-dimensional fiber coordinates were then divided by total cochlear length (i.e., distance from the most basal to the most apical fiber along the fiber tip trajectory) and used as *x* for the Greenwood equation (Equation 2). Note that in the original equation Greenwood defined *x* as the fraction of cochlear length with *x* = 1 at the basal end. As this is inverted to our definition, it was necessary to replace *x* in the original equation with (1−*x*), resulting in Equation 2.


(2) $$\begin{array}{r} CF=165.4\text{Hz}\cdot \left(10^{2.1\cdot \left(1-x\right)}-0.88\right) \end{array}$$

**Figure 5:**
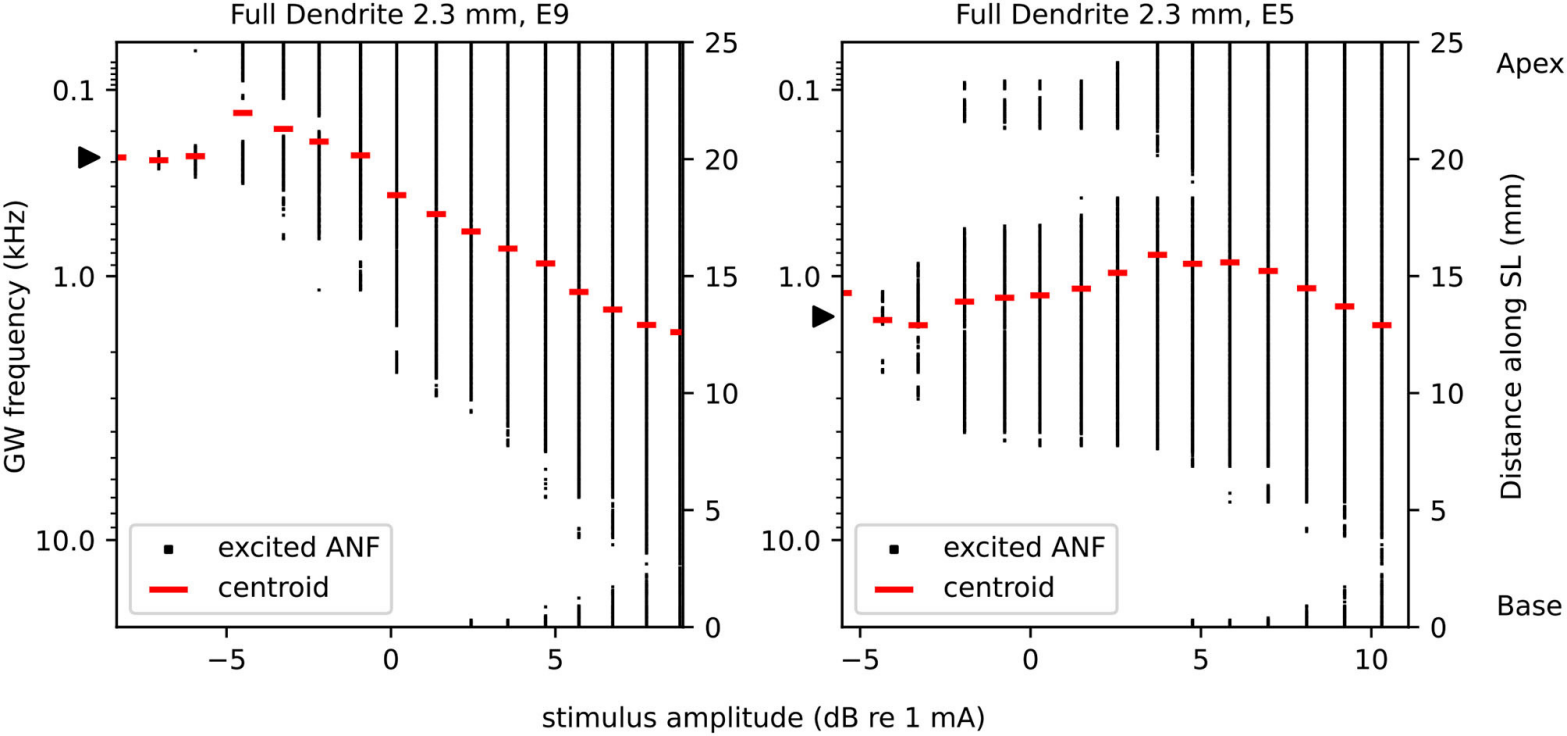
Excited fibers and corresponding centroids, exemplary for FD_2.3_ electrode 9 (left) and electrode 5 (right). Electrode 9 features a decrease and subsequent increase of pitch corresponding to the centroids with increasing stimulation amplitude. Electrode 5 features ectopic activation. For purpose of better visualization, we included here only a selective number of stimulation amplitudes. The electrode position is mapped to its nearest fiber, indicated by a black triangle. The left ordinate shows the frequency map, right ordinate the position of ANFs along the spiral lamina (SL).

In addition, Laneau et al. (2004) observed that a model predicting place pitch based on the centroid of the excited cochlear area fits well with the results from their pitch ranking experiments on CI subjects. Therefore, we employed excitation profiles to reconstruct an approximation of “perceived" place pitch, based on the centroid of excited fibers and the Greenwood frequency map. The centroids were computed as the mean of 1-dimensional fiber tip coordinates of only the excited fibers, for any individual stimulation current. An illustration describing centroid reconstruction and corresponding pitch is displayed in Figure 5. Note that it was not distinguished whether there was a gap in excited fibers (i.e., due to ectopic activation, e.g., Figure 5, electrode 5), which could potentially lead to a perception of two separate pitches instead of one. The reconstructed pitch was then further evaluated by computing the difference in pitch between adjacent electrodes, for four selected percentages (5%, 10%, 15%, and 20%) of EDR. Pitch difference was calculated in octaves, i.e., $log_{2}\left(\frac{F_{a}}{F_{b}}\right)$, with *F*_*a*_ being the pitch of the more apical electrode, and *F*_*b*_ being the pitch of the more basal electrode.

## 3. Results

### 3.1. Neural Excitation

#### 3.1.1. Absolute Threshold

**Figure 6:**
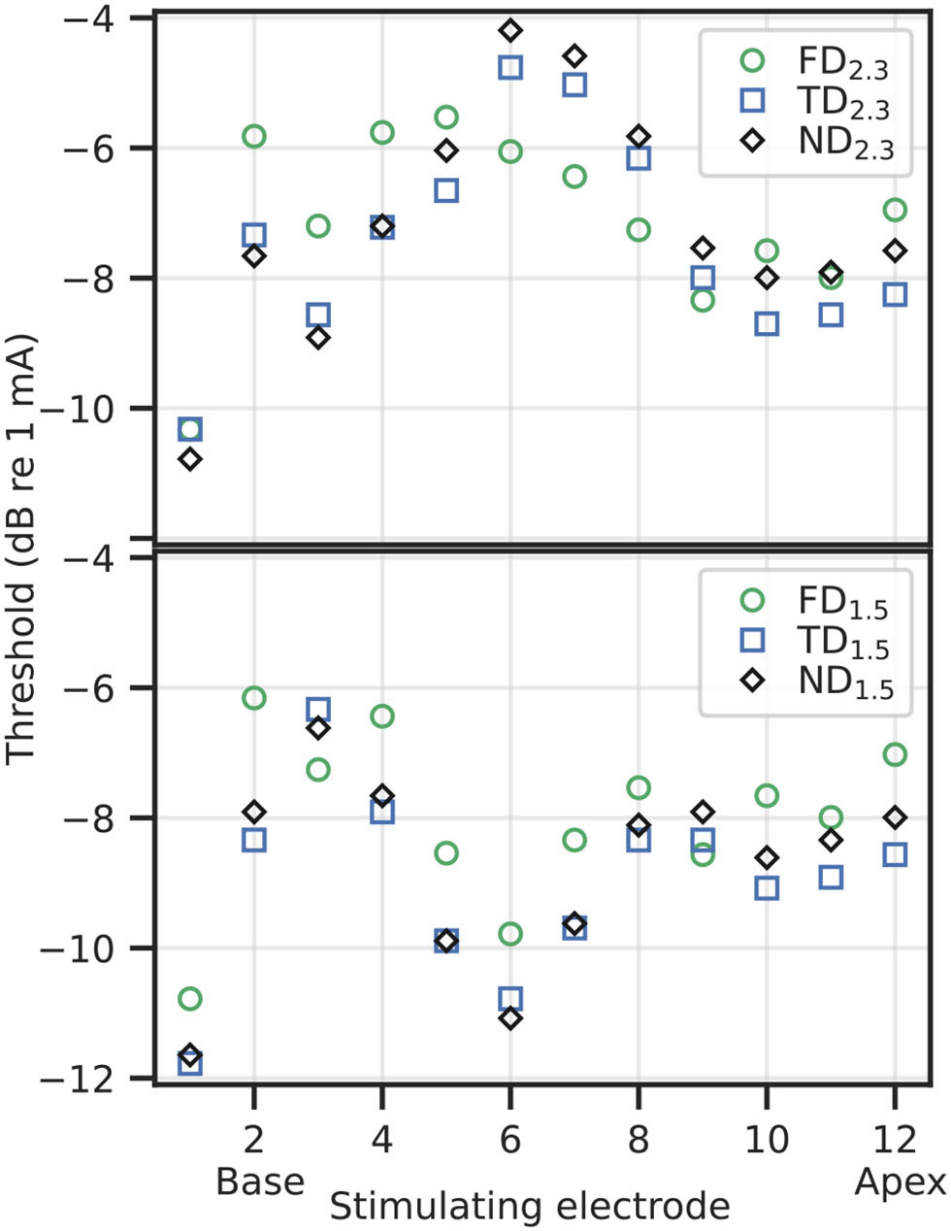
Thresholds for individual stimulation electrodes and degenerative states, 2.3 mm dendrite on top, 1.5 mm dendrite on bottom.

Absolute threshold refers to the lower limit of the EDR, i.e., the minimum current necessary to elicit an AP in at least one ANF in a simulated population. In Supplementary Figure S1, the voltage traces of selected ANFs at and just below the absolute threshold have been included. Absolute thresholds, as shown in Figure 6, ranged from approximately −12 dB re 1 mA (= 0.25 mA) to approximately −4 dB re 1 mA (= 0.63 mA). In general, dendritic degeneration lead to a slight reduction in the absolute threshold, which was unexpected. Compared to FD, absolute thresholds for TD and ND were reduced in base and apex for 2.3 mm dendrites, and over most of the cochlear length for 1.5 mm dendrites. Within individual stimulation electrodes, absolute thresholds of TD and ND were within approximately 2 dB difference compared to their FD counterpart, and within 1 dB difference when compared with each other. TD absolute thresholds were slightly below ND absolute thresholds in most cases.

#### 3.1.2. Excitation Profile

**Figure 7:**
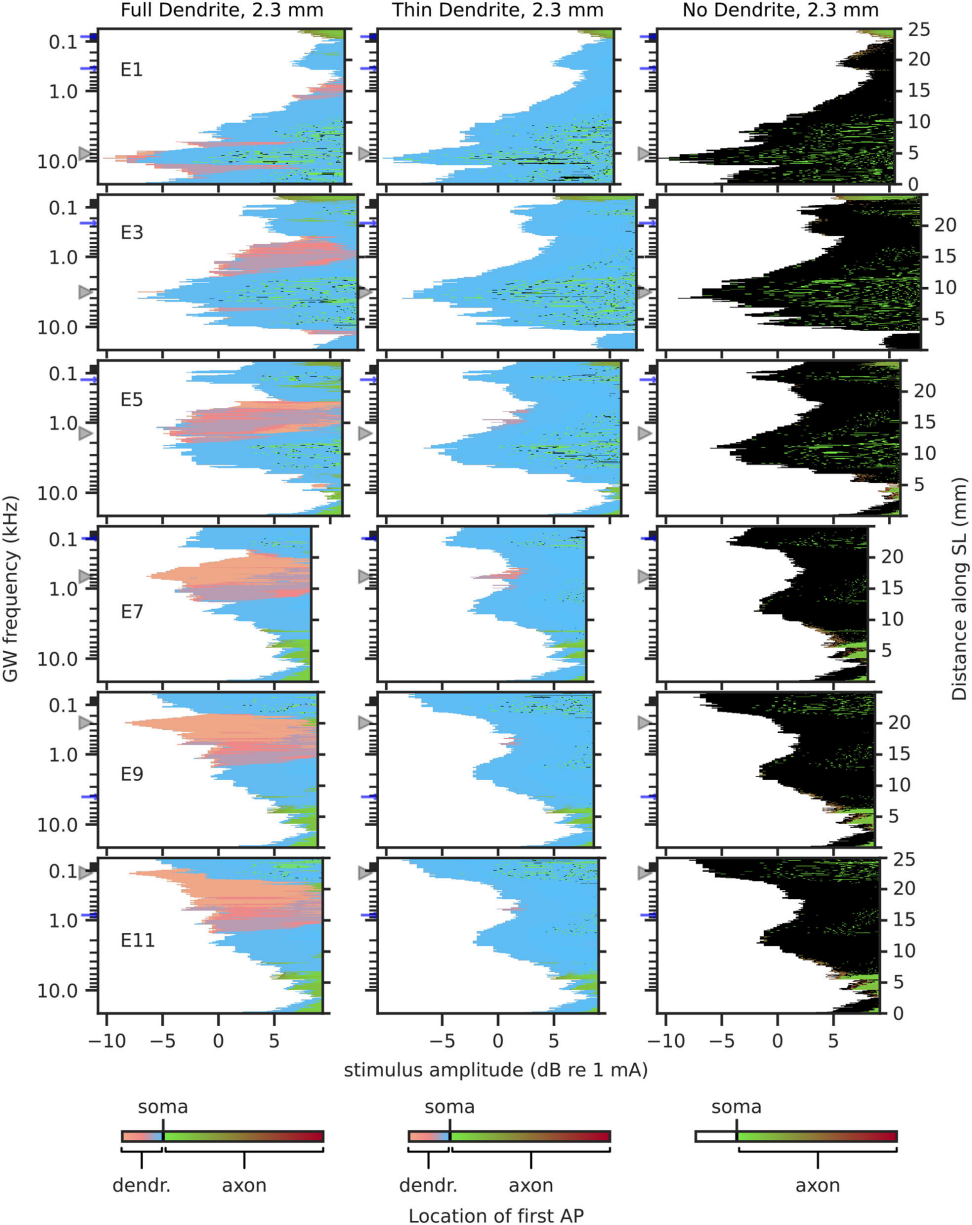
Excitation profiles for 2.3 mm dendrites. Greenwood frequency mapping performed for all 400 fibers, marked on the left ordinate. Fiber position along spiral lamina marked on the right ordinate. AP initiation site is color-coded, soma marked with black color. Stimulation electrodes labeled on the left. Electrode position is mapped to its nearest fiber and indicated by gray triangles, blue arrows mark fibers at ±360° to the stimulation electrodes.

**Figure 8:**
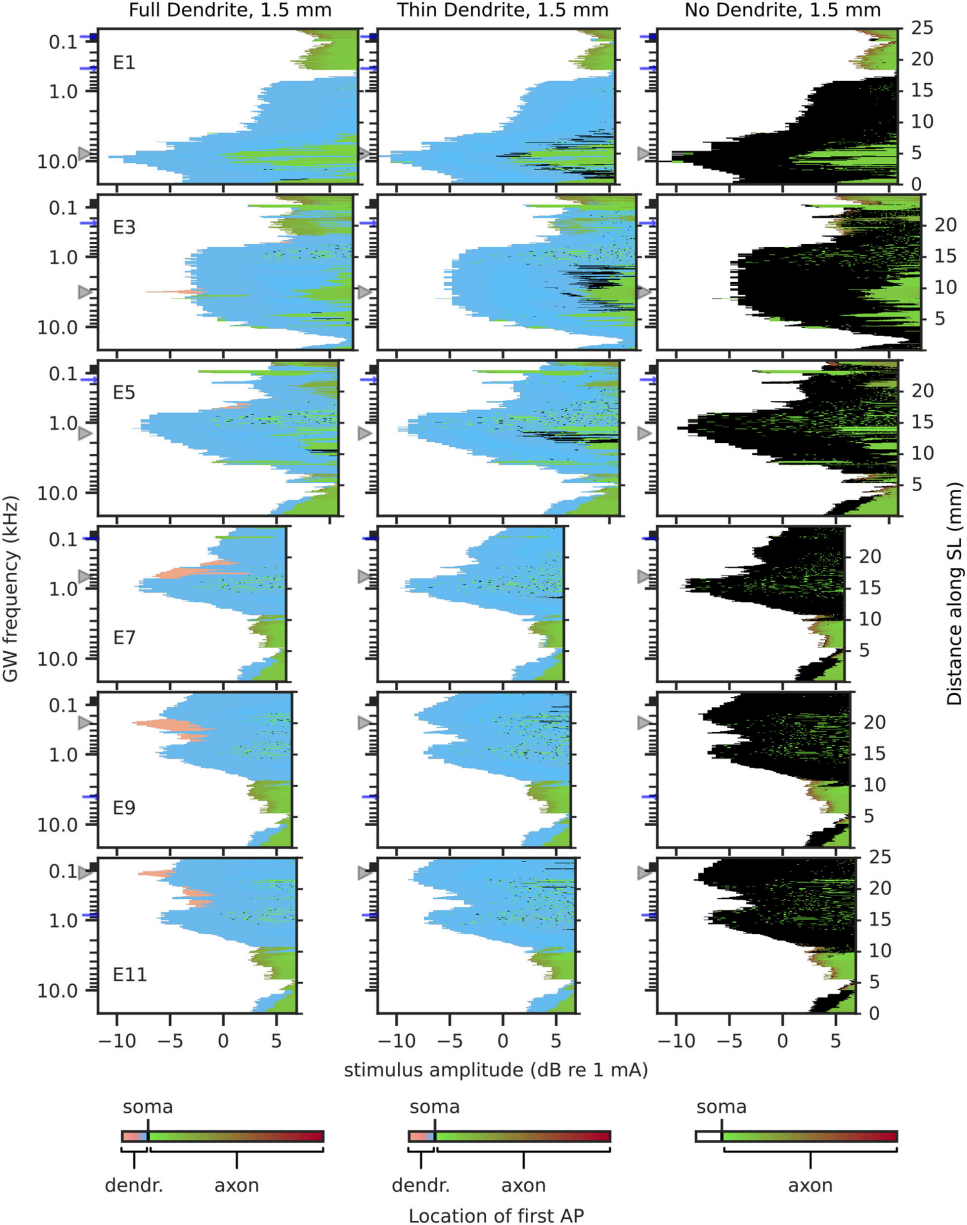
Excitation profiles for 1.5 mm dendrites. Greenwood frequency mapping performed for all 400 fibers, marked on the left ordinate. Fiber position along spiral lamina marked on the right ordinate. AP initiation site is color-coded, soma marked with black color. Stimulation electrodes labeled on the right. Electrode position is mapped to its nearest fiber and indicated by gray triangles, blue arrows mark fibers at ±360° to the stimulation electrodes.

Excitation profiles depict the stimulation amplitude at which each fiber was activated and include the location of initial “effective” excitation within each fiber. Hereby, the “effective” excitation refers to the excitation that produces an afferent AP. For example, if a neuron is first excited in its dendrite, but before the AP propagates to the axon, it is excited independently in the axon as well, then the initial “effective” excitation is determined to originate from the axon. Excitation profiles for 2.3 mm dendrites are shown in Figure 7, and for 1.5 mm dendrites in Figure 8. Ectopic activation was observed in the excitation profiles. Here, ectopic activation is defined as independent activation of regions distant to stimulating electrodes; for example in Figure 7 TD_2.3_ activation from electrode 7 at approximately 150 Hz and 2 kHz, as well as electrode 9 at approximately 30 Hz and 2 kHz.

For FD_2.3_ (Figure 7), it could be observed that AP initiation sites were mostly proximal to the soma, except for regions close to the electrodes, where AP initiation happened in the distal region of the dendrite, especially for middle and apical stimulation electrodes, but less so for basal stimulation electrodes. This may indicate that stimulation of neurons in the basal turn might be affected less by degeneration, as APs initiated in the somatic area are less likely to be influenced by changes in dendrite morphology. Ectopic activation could be observed especially for stimulation in the middle of the cochlea. Regarding TD_2.3_ and ND_2.3_, visual inspection showed very similar stimulation patterns between each other. The activation initiated in the distal region of the dendrites in the case of FD_2.3_ was largely missing in both degenerated cases, in spite of that dendrites were still present with TD_2.3_. The most striking difference between the two degenerated cases is that for TD_2.3_, most APs initiated just before the soma, while for ND_2.3_, most APs initiated in the soma. Excitation profiles for individual apical electrodes (electrodes 9 and upward) showed very similar patterns among each other, both for TD_2.3_ and ND_2.3_. Ectopic activation was very prominent for both TD_2.3_ and ND_2.3_, especially in the middle and apex, where in most cases individual fiber thresholds were lower for ectopic activation than for fibers near the stimulation electrodes (e.g., electrode 7, activation at approximately 150 Hz). In summary, degeneration of 2.3 mm dendrites increased ectopic activation, caused less to no stimulation in the distal region of the dendrite, and reduced differences between apical stimulation electrodes.

In contrast to 2.3 mm dendrites, 1.5 mm dendrites (Figure 8) had the vast majority of APs initiated proximal to the soma, even for FD_1.5_, and more APs initiated in the axon for stimuli at high amplitudes. FD_1.5_ showed some initiations in the distal region of dendrites in the apex, but far less than FD_2.3_. Overall, differences among FD_1.5_, TD_1.5_, and ND_1.5_ were small, except for the few distal dendrite activations in FD_1.5_. Ectopic activation was only prominent in the apex for all degenerative states (e.g., electrode 11, activation at approximately 1 kHz). Similar to TD_2.3_ and ND_2.3_, excitation profiles for TD_1.5_ and ND_1.5_ showed little difference between individual apical electrodes.

For ND, most APs were initiated in the soma. On closer inspection, the vast majority of APs initiated in the soma (approximately 93%) showed indistinguishable AP peak timings and highly similar potential values (average difference at approximately 0.01 mV) to corresponding postsomatic compartments, i.e., APs initiated simultaneously in both the soma and the postsomatic compartment (not shown). Simultaneous AP initiation in both the soma and postsomatic compartment may indicate an excitation that originated from the axon hillock or axon initial segment, the interfaces between soma and axon.

Considering results from short dendrites to be more accurate in the middle of the cochlea, and results from long dendrites to be more accurate in the base and apex, the largest impact of degeneration may be in the apex, causing prominent ectopic activation and a small difference in areas stimulated by different electrodes. Ectopic activation in the base and middle of the cochlea was mostly cross-turn activation, i.e., activation at ±360° to the electrode.

#### 3.1.3. Individual Threshold Differences

**Figure 9:**
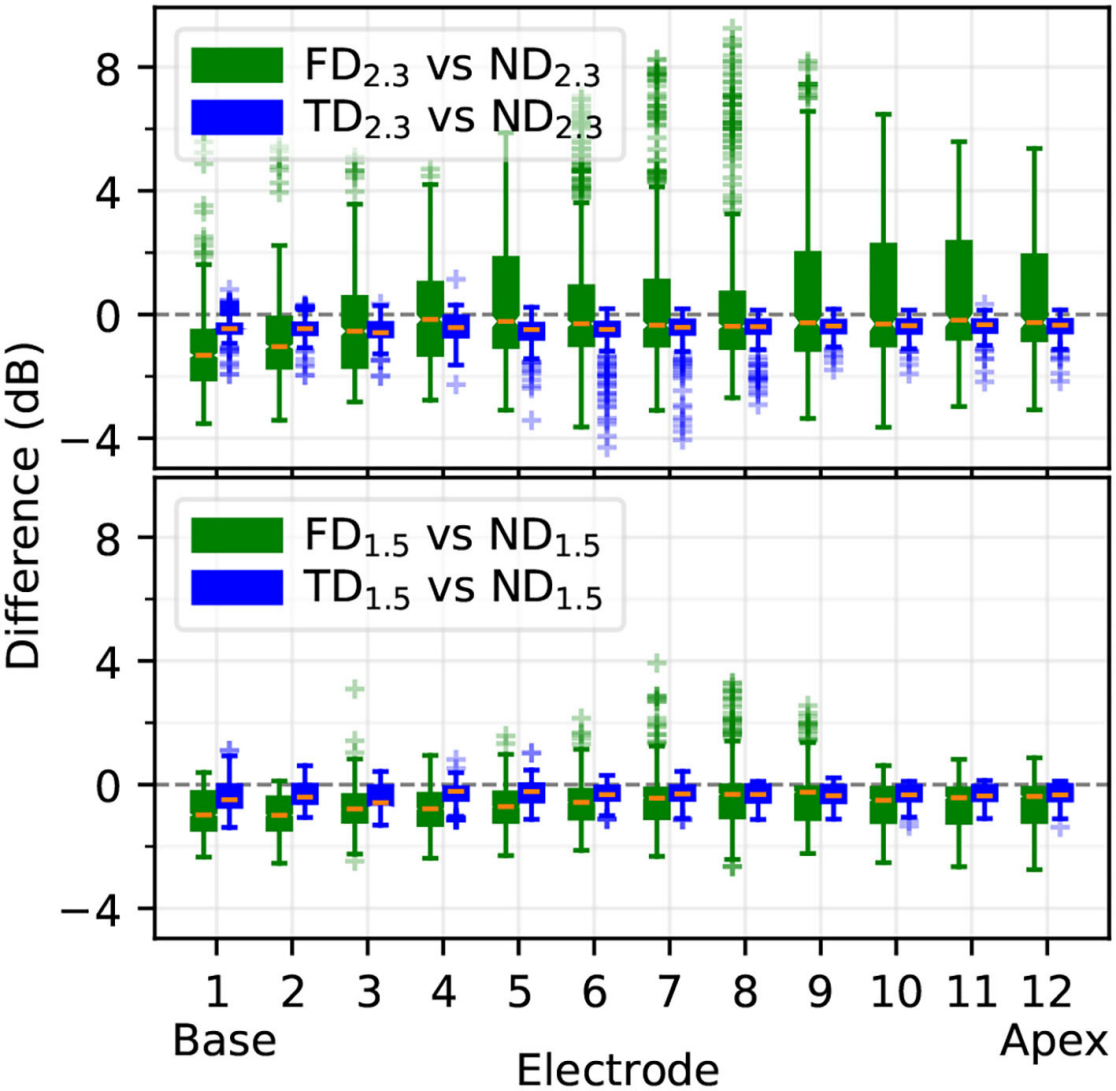
Difference of individual ANF thresholds between ND and other degenerative states, computed as in Equation 1. Boxplots show medians, lower and upper quartiles, whiskers (encompassing values contained in quartiles ± 1.5 times the interquartile range), and outliers.

Based on the excitation profiles, differences in individual fiber thresholds between degenerative states were computed, comparing how similar FD and TD were to ND. Differences (dB) are displayed in Figure 9. In all cases, ND was more similar to TD than to FD; the TD vs. ND difference median was on average at approximately –0.5 dB, with interquartile ranges of 1–2 dB. With 2.3 mm dendrites, electrodes in the middle showed outliers down to approximately –4 dB for TD_2.3_ vs ND_2.3_. FD_2.3_ vs. ND_2.3_ showed the largest differences, with difference medians from approximately -3 to 0 dB, and interquartile ranges of approximately 3 to 7 dB. FD_1.5_ vs. ND_1.5_ differences were larger than TD_1.5_ vs. ND_1.5_, but not as pronounced as for 2.3 mm dendrites. In general, differences between degenerative states of 1.5 mm dendrites were smaller than differences between degenerative states of 2.3 mm dendrites, and differences between TD and ND were much smaller than differences between FD and ND.

### 3.2. Pitch

**Figure 10:**
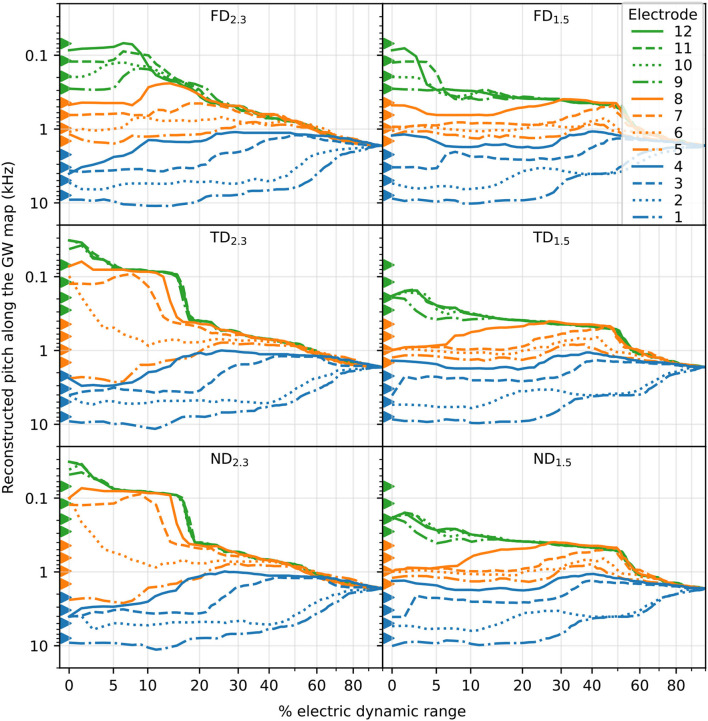
The reconstructed pitch along % of EDR. Pitch reconstruction was based on the centroid of the excited area and the Greenwood map. Basal, middle, and apical electrode stimulations are marked by color, and individual electrodes are marked by line style. Electrode position, corresponding to the pitch of its nearest fiber, is marked with triangles.

Pitch was reconstructed based on the centroid of excited area and the Greenwood frequency map (see also Figure 5). Reconstructed pitch for all electrodes and degenerative states along each corresponding EDR is displayed in Figure 10. At larger stimulus amplitudes (greater than approximately 20% EDR), pitch of apical electrodes largely overlapped. This partly applied to middle electrodes as well, especially for long dendrites. Below approximately 20% EDR, however, pitch for different electrodes differed depending on degenerative state and dendrite length, as detailed below. Note that for higher percentages of EDR, reconstructed pitch would automatically converge to approximately 1,700 Hz, corresponding to the middle of the cochlea. For example, with 50% of ANFs excited, the average excited position would always result within the innermost 50% of fibers (i.e., ANF 101 to ANF 300, for 400 ANFs), therefore the centroid of the excitation would correspond to a CF between approximately 400 Hz and 6 kHz. Consequently, with 100% of ANFs excited, the average excited position would always be at the middle of the cochlear length, corresponding to approximately 1700 Hz.

For FD, the pitch at the absolute threshold was mostly well spaced for all electrodes, with pitch usually evoked close to stimulating electrodes. With increasing stimulus, at approximately 10% EDR for FD_2.3_ and 5% for FD_1.5_, the pitch of apical stimulation electrodes increasingly overlapped. In contrast, for both TD and ND there were overlapping pitches even at the absolute threshold. There, for TD_2.3_ and ND_2.3_, electrodes 9–12 evoked pitch at approximately 30–40 Hz, which increased to approximately 80 Hz at 5% EDR. Note that the Greenwood frequency map with parameters suggested for humans starts at approximately 20 Hz (Greenwood, 1990). Evoked pitches of middle electrodes (5–8) were not close to any of the middle electrodes but instead offset toward either the apex (electrodes 6–8) or the base (electrode 5). For TD_1.5_ and ND_1.5_, all electrodes 9–12 evoked pitch at approximately 190 Hz (between electrodes 9 and 10) at the absolute threshold. No pitch was evoked close to electrodes 4, 7, 8, 9, 11, and 12 at the absolute threshold, with no pitch evoked close to electrodes 11 and 12 even with increased stimulus. The pitch evoked by basal stimulating electrodes was similar to FD pitch for all TD and ND. Yet another interesting observation was a few cases of pitch reversals, i.e., an apical electrode generating a higher pitch than a more basal electrode. Pitch reversals were seen with some apical electrodes at specific ranges of EDR, e.g., electrode 12 generated higher pitches than electrodes 9–11 for FD_2.3_ at approximately 10–25% of EDR.

With increased stimulation amplitude, abrupt changes in reconstructed pitch were often observed, especially for apical electrodes. This can generally be attributed to ectopic activation, where a new group of neurons is excited and thereby changes the overall pitch (e.g., TD_2.3_ electrode 7). In real CIs, however, this might cause a second, parallel pitch perception instead of a shift in pitch. In addition, while we investigated stimulation by individual, independent electrodes, in implanted CIs sounds will likely activate more than a single electrode, therefore a perceived pitch would be the outcome of a combination of multiple activated electrodes.

In summary, FD showed mostly independent and tonotopic frequency channels for different electrodes, especially near the absolute threshold, but TD and ND both lead to the unification of several frequency channels, especially for apical electrodes. TD and ND showed similar pitch values compared to each other. In some cases, pitch reversals were observed.

#### 3.2.1. Pitch Difference

**Figure 11:**
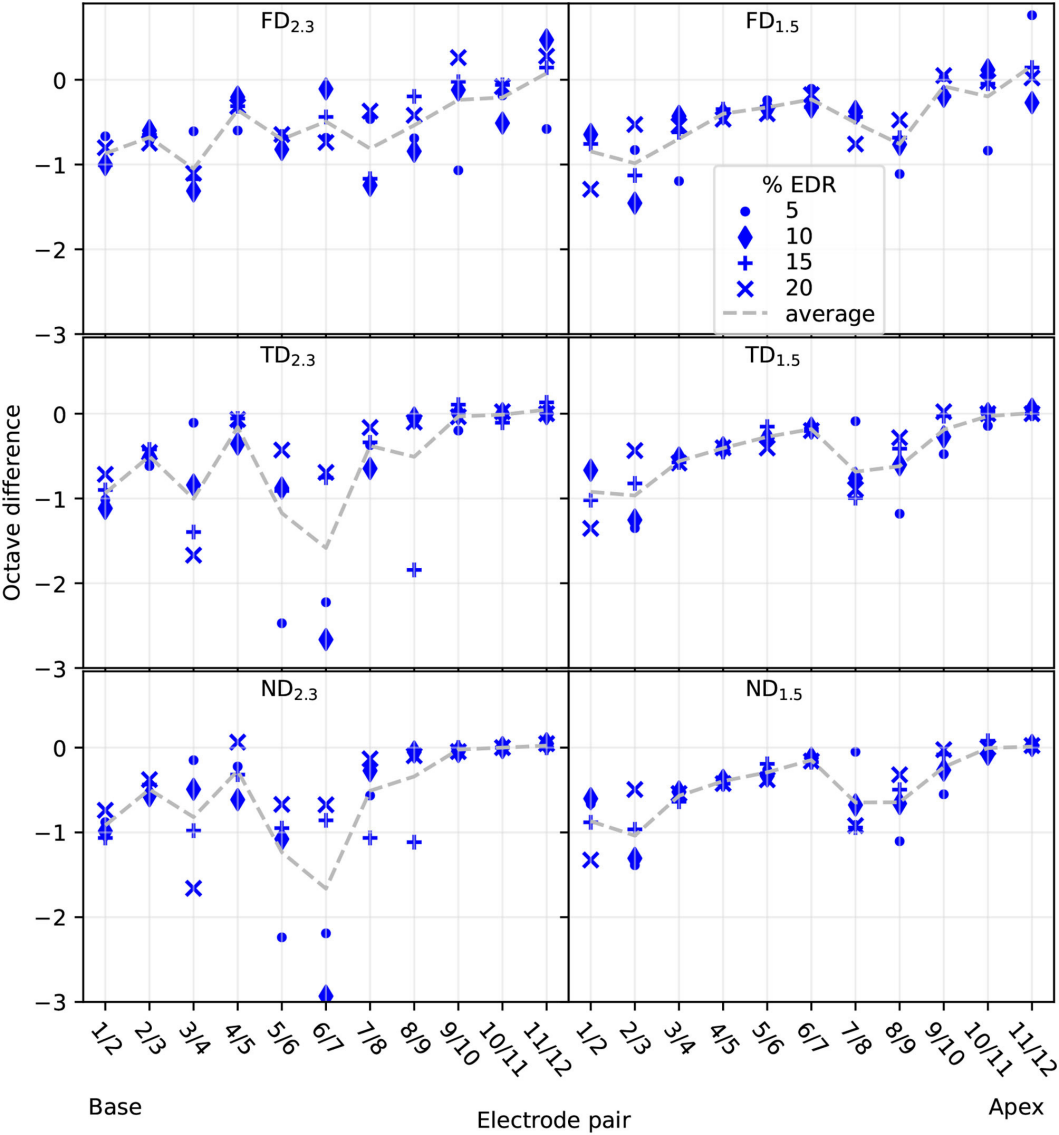
Reconstructed pitch difference between neighboring electrodes. Differences displayed for 5, 10, 15, and 20% of EDR, as well as the average of the four differences. Electrode pair refers to which electrodes were used to compute the difference.

Pitch difference, depicted in Figure 11, was mostly in the 0 to −1 octave range. All configurations showed almost no differences for apical electrodes when averaged over the four selected percentages. However, note that for FD this was the case because of pitch reversals occurring, and differences in individual EDR percentages were mostly non-zero. In contrast, TD and ND showed almost no differences in the apex not only for the averaged values but for most of the individual EDR percentages as well, indicating similar pitches evoked by different apical electrodes over an extended current range. This was especially prominent for TD_2.3_ and ND_2.3_, where differences were close to zero octaves for electrodes 8–12. TD_1.5_ and ND_1.5_ differences were close to zero octaves only for electrodes 10–12. Differences for TD_2.3_ were similar to differences for ND_2.3_, and differences for TD_1.5_ were similar to differences for ND_1.5_.

## 4. Discussion

The present study investigated CI excitation of ANFs with different degrees of degeneration, based on morphological measurements on human SGN dendrites and a high resolution FE model of a human cochlea. Recent measurements indicated that intact human SGN dendrites possess a diameter of approximately 2.0 μm and that degeneration of SGNs may also manifest in a reduced diameter of approximately 0.5 μm (Heshmat et al., 2020). The cochlea FE model was based on μCT scans of a human cochlea and, therefore, generated a highly personalized model, including fine details of cochlear structures down to the porous structure of the modiolar bone (Bai et al., 2019). The cochlea model was implanted with a MED-EL Standard electrode array consisting of 12 electrode pairs and stimulated in a monopolar configuration with biphasic pulses. Potential distributions were obtained from the FE model and used as a stimulus for a large population of Hodgkin-Huxley-based biophysical multi-compartment neuron models adapted to represent human ANFs (Rattay et al., 2013), spread out along the entire cochlea. Primary simulation results consisted of detailed excitation profiles covering both cochlear length and electric dynamic range for all 12 electrodes of the implant.

Studies modeling retrograde degeneration of ANFs commonly modeled degeneration by removing either the entire dendrite or peripheral parts of it (Rattay et al., 2001, 2013; Briaire and Frijns, 2006; Smit et al., 2008, 2010; Snel-Bongers et al., 2013; Kalkman et al., 2014; Malherbe et al., 2015; Potrusil et al., 2020). This study included an “intermediate" degenerative state as well, a thin dendrite with only a 0.5 μm diameter, based on measurements from Heshmat et al. (2020). A shrinkage of dendrite diameter in the process of degeneration was also observed by Wise et al. (2017) in guinea pigs, though only manifesting in reduced diameter of the axoplasma, but not the surrounding myelin sheath. Differences between myelin sheath measurements may be due to examinations performed on different species, with Heshmat et al. measuring humans and Wise et al. measuring guinea pigs. Our study modeled axon and myelin sheath diameter according to Heshmat et al. (2020). In addition, we modeled homogeneous degeneration in the entire cochlea. This is likely not the case in real implanted cochleae, where there may be heterogeneous degeneration. For example, assuming that hearing loss correlates with degeneration of ANFs since age-related hearing loss generally is more pronounced in high frequencies (Brant and Fozard, 1990; Wiley et al., 2008), it could be concluded that basal ANFs are more likely to degenerate than apical ANFs, at least for most aged implanted subjects. Nevertheless, investigation of different degeneration patterns in the cochlea was beyond the scope of this study. Surprisingly, results were largely identical between thin dendrite and no dendrite states, especially in contrast to intact dendrite state, for all investigated parameters, including absolute and individual thresholds, excitation profiles, and reconstructed pitches. This may be attributed to the diameter of 0.5 μm being already very close to failing to be able to transmit APs through the soma when modeled as a deterministic model (<0.4 μm, Heshmat et al., 2020), leading to a largely “inactive” dendrite in terms of neuronal excitation. As the diameter of 0.5 μm was picked to represent one of two peaks in a quantity distribution, with the other peak being 2 μm, representing “intact” dendrites, simulating an additional diameter other than the two values would be arbitrary. Therefore, it may be concluded that simulation of ANF degeneration by only removal of dendrites is sufficiently accurate. An important constraint to this conclusion, however, is that other cochleae and stimulation configurations (i.e., monopolar vs. multipolar, alternate pulse shape) may show larger amounts of excitation in the distal part of the dendrite for TD than observed in our investigation, where we considered only monopolar biphasic stimulation. Therefore, multipolar stimulation might increase the difference between TD and ND, making the simulation of intermediate degenerative states necessary.

Absolute thresholds for individual electrodes were defined as the current necessary to excite at least a single ANF of a population. Intuitively, one would expect that with increasing degeneration absolute thresholds would increase as well, as the nearest part of an ANF to stimulating electrodes in lateral electrode arrays is generally the peripheral terminal (Stakhovskaya et al., 2007; Mistrík et al., 2017). Therefore, degeneration would increase the electrode-neuron distance, which is often attributed proportionality to the perceptual threshold (Pfingst and Xu, 2004; Goldwyn et al., 2010; Long et al., 2014; Bierer et al., 2015). In this study, however, not only an absolute threshold increase with degeneration was observed, but also an absolute threshold decrease. This appears counterintuitive only at first glance, however, when inspecting AP initiation sites, it could be observed that the absolute threshold was reduced with increased degeneration whenever fibers were initially excited proximal to the soma for FD. This could be explained using the intracellular resistance *R*_*a*_ of the dendrite: if the dendrite is reduced in diameter or removed, dendritic *R*_*a*_ is increased. More specifically, from FD to TD resistance would increase by a factor of 16 (approximately 1.6 MΩ for 10 μm length of dendrite with 2.0 μm diameter, and approximately 25.5 MΩ for 10 μm length of dendrite with 0.5 μm diameter), and for ND resistance would essentially increase to ∞Ω ($R_{a}=\frac{resisitivity\cdot length}{radius^{2}\cdot \pi }$). Such an increase in dendritic intracellular resistance would lead to less or no outward current flow from the soma toward the dendrite, therefore facilitating the charging of cell membranes in the somatic area when compared to a neuron with a thicker dendrite. Therefore, whenever APs at the absolute threshold were initiated in or at the soma for FD, this effect would manifest in a reduction of the absolute threshold for TD and ND. Conversely, this did not apply in all cases, however. For absolute thresholds where APs originally initiated in the distal end of the dendrite, absolute thresholds tended to increase with degeneration. However, there were exceptions where thresholds would still decrease, e.g., electrode 11 as shown in Figure 7, where ectopic activation initiated at the soma for low currents already and compensated for threshold increase of ANFs near the electrode. Threshold differences between degenerated states TD and ND were small, with ND mostly displaying slightly higher absolute thresholds. This could be attributed to APs in ND being mostly initiated at the interface between soma and axon, which appears to be less “optimal” than AP initiation directly before the soma.

While excitation of ANFs in natural hearing originates from inner hair cells, and therefore, excites ANFs at their peripheral terminal, excitation of ANFs under CI stimulation has been shown to happen at several distinct sites along the fibers (Miller et al., 2003). Suggested initial excitation sites are peripheral terminal, dendrite, and axon in general (Rattay et al., 2001; Briaire and Frijns, 2006; Kalkman et al., 2014; Potrusil et al., 2020), as well as areas proximal to somata (Cartee, 2006; Cartee et al., 2006). In general, APs in our model were initiated at one of three “preferred" sites: the distal end of the dendrite, compartments directly peripheral to the soma, or the interface between soma and axon. Only for very large stimuli (>10 dB re absolute threshold) extended initiation in midst of the axon was observed, where stimulation may very well be above maximum loudness levels acceptable for CI subjects (dynamic ranges of patients with CI are on average at approximately 10 dB for low pulse rates Kreft et al., 2004; Zhou et al., 2012).

Our results showed AP initiation in the distal end of the dendrite happened only in areas near stimulation electrodes, and for the most part only in the apex and when dendrites were intact (FD), given that short dendrites hardly had any AP initiation in the distal end of dendrites for electrodes in the middle of the cochlea. Overall, initiations in the distal part of the dendrite and the axon were the minority, especially for degenerated dendrites (TD and ND). Therefore, most APs initiated in the somatic area, either directly before the soma (TD) or at the interface between soma and axon (ND). In terms of excitation profile shape, differences were the smallest between TD and ND. Except when TD was initiated in the distal end of the dendrite, the same currents generally excited the same neurons for both TD and ND. Initial excitation in the distal end of the dendrite for TD happened only in a minuscule number of neurons in the middle of the cochlea and in no case at the absolute threshold.

Ectopic stimulation, including cross-turn stimulation, is both seen in modeling studies (Frijns et al., 2001; Hanekom, 2001; Briaire and Frijns, 2006; Kalkman et al., 2014) and discussed or suspected in patient studies (Frijns et al., 2002; Arnoldner et al., 2008; Finley et al., 2008). Our results showed prominent ectopic activation in the excitation profiles, especially for degenerated dendrites and apical regions. Most importantly, for apical stimulation electrodes and degenerated dendrites, the same neuronal regions were excited preferentially, essentially independent of the stimulation electrode and likely generating an identical pitch perception. Ectopic activation manifested as both cross-turn activation (e.g., Figure 7 electrode 5), and non-cross-turn activation, i.e., neurons activated at an offset position to the stimulation electrode, but not at a distance of an entire turn (e.g., Figure 7 electrode 9). The existence of ectopic activation of less than 360° to the electrode position is less intuitive than cross-turn activation but has been mentioned in literature before, e.g., being suspected as the underlying cause for “tip-shifts" in forward masking spatial tuning curves with CI patients by Nelson et al. (2008). In summary, ectopic activation especially occurred for degenerated ANFs, mainly impacting stimulation in the apex. Stimulation in the base and middle of the cochlea showed less ectopic activation for degenerated dendrites than in the apex, assuming longer (2.3 mm) dendrites in the base and apex and shorter (1.5 mm) dendrites in the middle of the cochlea. This was likely due to most APs initiating near the soma with FD in the basal and middle turns. In contrast, with FD most activation in the apex happened at the peripheral terminal for ANFs near the stimulation electrodes, which was absent with degenerated neurons.

Excitation profiles give a good overview of the way the ANF population is excited, but given that the purpose of a CI is to elicit sound perception, such profiles are still abstract. In order to approximate the perception of the simulated results, stimulated neurons were mapped to place pitch using the centroid of the excited area (Laneau et al., 2004) and Greenwood's frequency map (Greenwood, 1990). Frequency mapping was performed for the entire EDR. However, not the entire EDR is relevant, but only the interval between perceptual threshold and maximum comfortable loudness of CI patients. Maximum comfortable loudness was arbitrarily estimated to correspond to 4 mm of neurons excited along together with the organ of Corti by Briaire and Frijns (2006). With an average Organ of Corti length of approximately 33 mm (Stakhovskaya et al., 2007), this would correspond to approximately 10% of fibers excited. For this reason, the reconstructed pitch was only inspected in detail for low percentages of EDR, up to approximately 20% of EDR. Results showed that for FD, individual stimulation electrodes generated individual pitches for low percentages of EDR, albeit less so for apical electrodes. With degenerated dendrites, however, pitches obtained for apical stimulation electrodes (especially electrode 9 upwards) were very similar, which essentially meant that different electrodes evoked the same pitch perception. With electrode 9 positioned at 450° insertion into the cochlea, it indicates that insertion of cochlear electrode arrays deeper than approximately 450° may not provide any benefit when ANFs are degenerated. A similar observation was drawn from the model of Kalkman et al. (2014), where electrodes beyond approximately 540° insertion stimulated nearly identical regions in the cochlea when ANFs were degenerated. Increased pitch confusion in the apex, i.e., the inability to correctly rank electrodes according to their pitch, was also observed in patient studies with deeply inserted electrodes (Deman et al., 2004; Gani et al., 2007; Landsberger et al., 2014). For example, Deman et al. observed that for CIs with electrode insertion depth between 471 and 662° about half of their subjects had similar pitch percepts for apical stimulation electrodes in pitch ranking experiments. Consequently, based on our results that degenerated ANFs lead to highly similar pitches in the apex, increased pitch confusion for deeply inserted apical electrodes could be an indicator of the neural health of apical neurons. In addition to similar reconstructed pitches for apical electrodes, our results showed pitch reversals in some cases. Pitch reversals are defined as pitch elicited from a more apically located electrode being perceived as higher than pitch elicited from a more basally located electrode, despite more apical positions corresponding to lower frequencies on the tonotopic map of the cochlea. Pitch reversals have been occasionally observed in psychophysical experiments as well, e.g., Fielden et al. (2015).

In conclusion, “intermediate” degenerative states of ANF dendrites in our model generally showed very similar effects to completely degenerated ANF dendrites. The largest impact of degeneration is expected in the apex, where different electrodes stimulate the same neurons for degenerated ANFs. Therefore, a deep insertion of electrode arrays into the cochlea may not provide any benefits for patients with degenerated ANFs. However, the impact of degeneration for electrodes up to approximately 450° insertion angle may be small. Nevertheless, it is important to note that this observation is based on monopolar biphasic stimulation of a single human cochlea. Multipolar stimulation is expected to be more heavily impacted by neural degeneration (Goldwyn et al., 2010; Zhu et al., 2012; Long et al., 2014; George et al., 2015; Heshmat et al., 2021), and different pulse-shapes are believed to preferentially stimulate different parts of an ANF (Rattay et al., 2001), which may increase the impact of degeneration and induce more differences between TD and ND. In addition, given that performance of patients with CI often varies between individuals, we can not make any judgment on how well the subject on whose temporal bone our cochlea model was based would have performed with a CI. However, we are currently working on building an extended set of FE models based on additional high-definition scans of human temporal bones. Therefore, the current investigation into specific SGN morphologies presents a starting point for highly personalized models which take into consideration the influence of fine details of cochlear structures. This will be expanded in future studies building on the current work, including not only additional cochleae but also investigations into further stimulation configurations and SGN morphologies. Therefore, using a larger number of high resolution cochlea models, we will investigate possible diagnostic methods for neural health in CI subjects, and whether the impact of degeneration on CI stimulation can be estimated based on preoperative measurements (e.g., cochlea shape from CT scans), possibly aiding in the selection of CI electrode design and insertion depth.

## Data Availability Statement

The datasets presented in this study can be found in online repositories. The names of the repository/repositories and accession number(s) can be found below: https://gin.g-node.org/Croner/dendritic_sgn_degeneration.

## Author Contributions

AC conducted the simulations and contributed to study design, ANF cable model implementation, data analysis, visualization, and manuscript writing. AH contributed to study design, SGN morphology measurements, and manuscript revision. AS-F contributed to study design, SGN morphology measurements, funding acquisition, and supervision. RG contributed to SGN morphology measurements and manuscript revision. WH contributed to study design, data analysis, manuscript revision, funding acquisition, and supervision. SB contributed to study design, FE model reconstruction, ANF path reconstruction, manuscript revision, and supervision. All authors contributed to the article and approved the submitted version.

## Funding

This project was funded by the Deutsche Forschungsgemeinschaft (DFG, German Research Foundation)–Projektnummer 415658392, the Austrian Science Fund (FWF) Grant No. l4147-B, and within the project “Modeling the electrical stimulation of the human cochlear nerve based on segmented high-resolution micro-computer tomography scans” under the D-A-CH program.

## Conflict of Interest

The authors declare that the research was conducted in the absence of any commercial or financial relationships that could be construed as a potential conflict of interest.

## Publisher's Note

All claims expressed in this article are solely those of the authors and do not necessarily represent those of their affiliated organizations, or those of the publisher, the editors and the reviewers. Any product that may be evaluated in this article, or claim that may be made by its manufacturer, is not guaranteed or endorsed by the publisher.
