# Supplementary material for: Effects of Degrees of Degeneration on the Electrical Excitation of Human Spiral Ganglion Neurons Based on a High-Resolution Computer Model
Source: Front Neurosci. 2022 Jul 6;16:914876. doi: 10.3389/fnins.2022.914876 (PMC9298973; doi:10.3389/fnins.2022.914876)
Supplement: Supplementary file 1 [file Data_Sheet_1.PDF]

Table S1. Parameter values of the model

**Parameter**	**Value**
**Finite element model**	
Electrode-electrode distance	approx. $2.4\,\mathrm{mm}$ $^{(1)}$
Electrode contact pad radius	approx. $0.18\,\mathrm{mm}$ $^{(1)}$
Scalp conductivity	$0.33\,\frac{\mathrm{S}}{\mathrm{m}}$ $^{(1)}$
Bone conductivity	$0.013\,\frac{\mathrm{S}}{\mathrm{m}}$ $^{(1)}$
Brain conductivity	$0.2\,\frac{\mathrm{S}}{\mathrm{m}}$ $^{(1)}$
Silicon electrode conductivity	$0\,\frac{\mathrm{S}}{\mathrm{m}}$ $^{(1)}$
Cochlear canal conductivity	$1.43\,\frac{\mathrm{S}}{\mathrm{m}}$ $^{(1)}$
Auditory nerve conductivity	$0.3333\,\frac{\mathrm{S}}{\mathrm{m}}$ $^{(1)}$
**Neuron model**	
Dendrite diameter	$2.0\,\mu\mathrm{m}$, $0.5\,\mu\mathrm{m}$
Axon diameter	$4.0\,\mu\mathrm{m}$
Soma diameter	$20\,\mu\mathrm{m}$ $^{(2)}$
Node of Ranvier length	$2.5\,\mu\mathrm{m}$ $^{(3)}$
Presomatic region length	$100\,\mu\mathrm{m}$ $^{(3)}$
$2.3\,\mathrm{mm}$ dendrite internode lengths	[305, 415, 415, 415, 415, 225] $\mu\mathrm{m}$ $^{(3)}$
$1.5\,\mathrm{mm}$ dendrite internode lengths	[305, 415, 415, 225] $\mu\mathrm{m}$
Axon internode lengths	$500\,\mu\mathrm{m}$ $^{(2)}$
Myelin thickness dendrite	$0.6\,\mu\mathrm{m}$ (71 layers), $0.15\,\mu\mathrm{m}$ (18 layers)
Myelin thickness axon	$0.68\,\mu\mathrm{m}$ (80 layers) $^{(3)}$
Membrane capacitance per layer	$1\,\frac{\mu\mathrm{F}}{\mathrm{cm}^2}$ $^{(3)}$
Membrane conductivity per layer	$1\,\frac{\mathrm{mS}}{\mathrm{cm}^2}$ $^{(2)}$
Maximum sodium conductivity (ion channels)	$120\,\frac{\mathrm{mS}}{\mathrm{cm}^2}$ $^{(3,\,4)}$
Maximum potassium conductivity (ion channels)	$36\,\frac{\mathrm{mS}}{\mathrm{cm}^2}$ $^{(3,\,4)}$
Maximum leak conductivity (ion channels)	$0.3\,\frac{\mathrm{mS}}{\mathrm{cm}^2}$ $^{(3,\,4)}$
Intracellular resistivity	$50\,\Omega\mathrm{cm}$ $^{(3)}$

Parameter values for the model implemented in this study. Values obtained from previous studies are marked by superscript:
$^{(1)}$: Values from Bai et. al. (2019)
$^{(2)}$: Values from Rattay et. al. (2013)
$^{(3)}$: Values from Rattay et. al. (2001)
$^{(4)}$: Conductivity is multiplied by ten for all active compartments aside of soma, as in Rattay et. al. (2001).

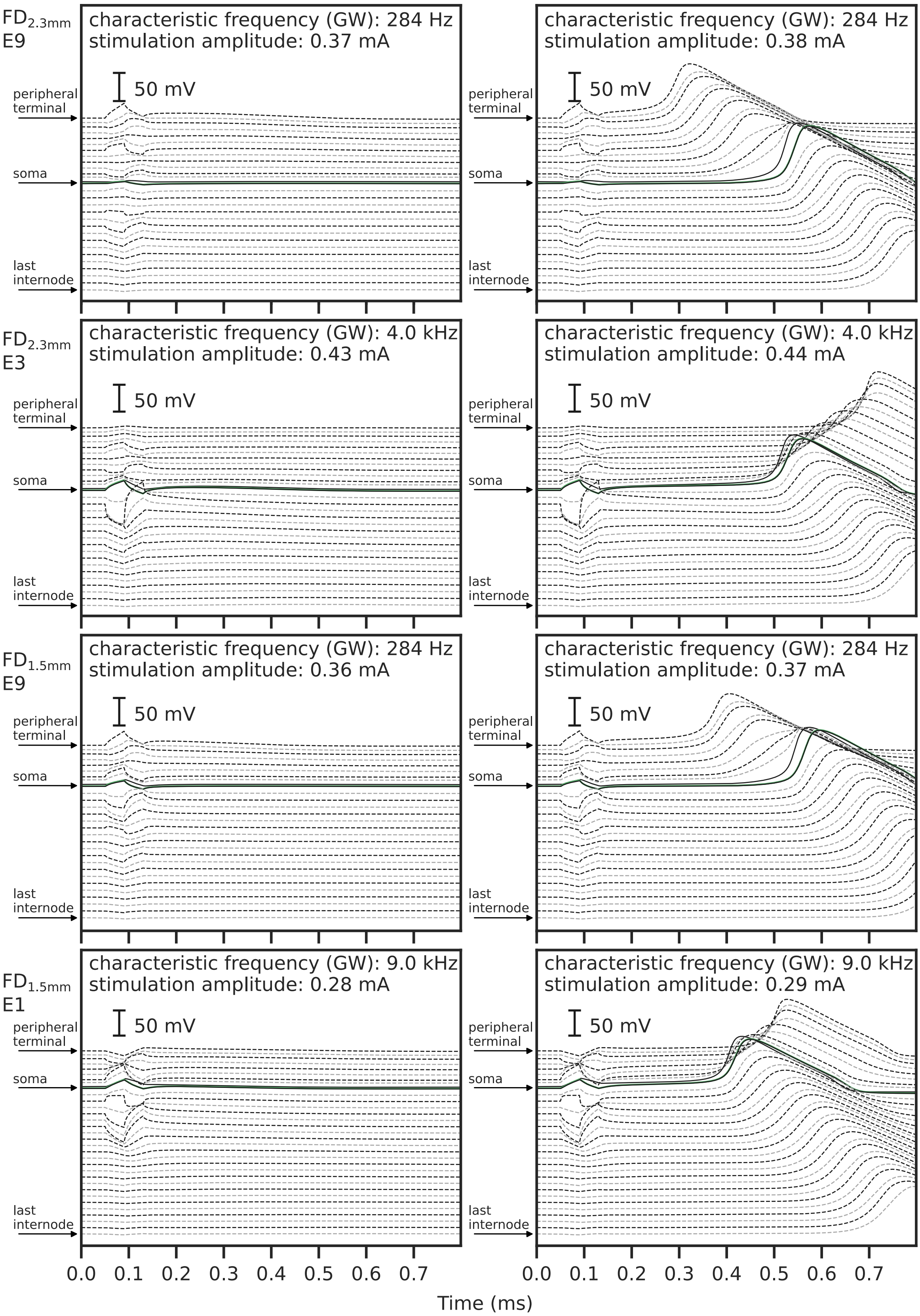

Figure S1. Voltage traces for selected fibers just below (left column) and at absolute threshold (right column). Fibers are indicated by their characteristic frequency (Greenwood map). All selected fibers are with fully intact dendrites. On the top left of each row, dendrite length is indicated by subscript, and stimulation electrode by number (e.g. E9 = stimulation electrode 9). Voltage traces of somata are displayed with green continuous lines, of pre- and postsomatic regions with black continuous lines, further active compartments with black dashed lines, and internodes with grey dashed lines.
